# Successful Treatment of Primary Eyelid Lymphedema by Periorbital Lymphovenous Anastomosis: A Case Report

**DOI:** 10.1055/s-0044-1792168

**Published:** 2024-12-27

**Authors:** Han Gyu Cha, Dong Yun Hyun, Eun Soo Park, Chang Yong Choi, Seung Min Nam

**Affiliations:** 1 Department of Plastic and Reconstructive Surgery, Soonchunhyang University Bucheon Hospital, Soonchunhyang University College of Medicine, Bucheon, Korea

**Keywords:** lymphedema, lymphovenous anastomosis, eyelid lymphedema, facial lymphedema, lymphovenous bypass

## Abstract

Eyelid lymphedema is a rare condition that presents as persistent swelling and non-pitting edema of the eyelids. Treatment options for this disease are limited, including surgical debulking and medications, which do not achieve complete resolution. Few studies have demonstrated the use of lymphovenous anastomosis (LVA) in the preauricular area for eyelid lymphedema treatment. In this report, we demonstrate the successful treatment of primary eyelid lymphedema by performing multiple LVAs in the periorbital region, where dermal backflow was visualized using indocyanine green lymphography. A total of four LVAs were performed through two separate incisions at the lateral canthal area and lateral eyebrow in a patient with unilateral upper eyelid lymphedema that resulted in significant improvement without recurrence.

## Introduction

Eyelid lymphedema is a rare condition that involves the upper or lower eyelid and presents as a persistent, non-tender swelling and non-pitting edema. Most reported cases of eyelid lymphedema are iatrogenic facial lymphedema secondary to head and neck cancer treatment or immunosuppressant administration. 1 2 Chronic eyelid lymphedema may also present as a typical symptom in patients with rare diseases, including Morbihan disease also known as rosacea lymphedema, and Melkersson–Rosenthal syndrome. 3 4 Therefore, eyelid lymphedema is generally diagnosed only after ruling out other diseases that present similar symptoms followed by histopathological confirmation. In histology, it is characterized by dermal edema, lymphangiectasis, and perivascular chronic inflammatory infiltration regardless of etiology. 5 6

Most eyelid lymphedema persists with aesthetic and functional concerns but no definite treatment options are available. Surgical debulking procedures including blepharoplasty have been mainly applied in many cases but resulted in unfavorable outcomes and recurrence. 2 7 8 Medical treatments also resulted in poor responses. 9 10

Here, we report a case of primary upper eyelid lymphedema that was successfully treated through multiple lymphovenous anastomoses (LVAs) in the periorbital region.

## Case

**Fig. 1:**
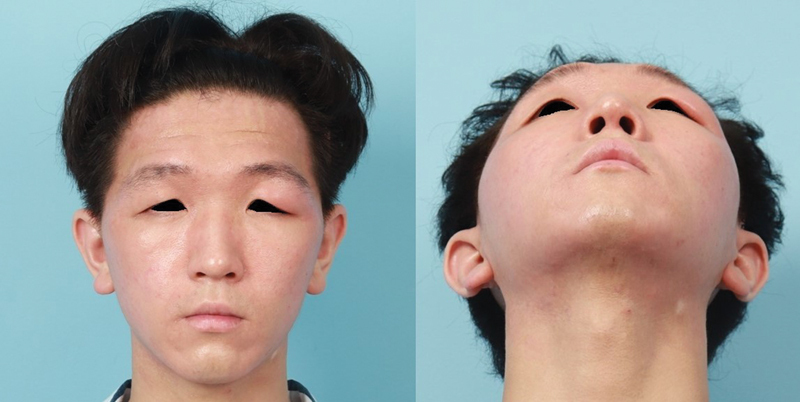
Clinical photographs of a patient with left upper eyelid lymphedema. A hard and non-tender swelling was seen on the left lateral upper eyelid and extended to the lateral canthus and lateral eyebrow.

A 22-year-old male visited the clinic with lymphedema in his left upper eyelid that began spontaneously 3 years ago. The lesion was located on the left lateral upper eyelid and extended to the lateral canthus and the eyebrow ( Fig. 1 ). It was hard, thickened, and showed non-tender swelling with redness and dry skin turgor. He was diagnosed with primary lymphedema at another hospital based on a punch biopsy. He had a history of surgical closure for a ventricular septal defect in the neonatal period and current atopic dermatitis on his face and neck.

**Fig. 2:**
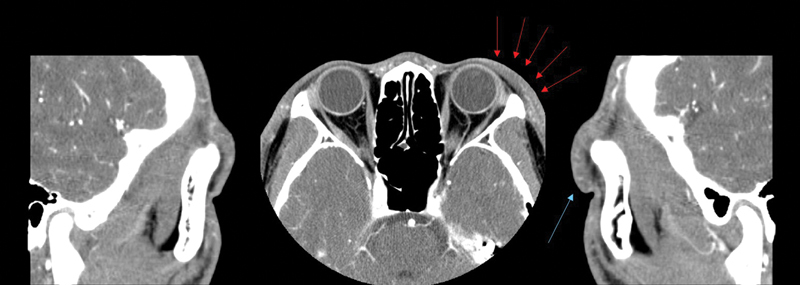
Facial computed tomography scan of a patient with left upper eyelid lymphedema. (Left) Sagittal view of unaffected side without any lesion. (Center) Axial view and (right) sagittal view of the affected side showing diffuse swelling compared to the contralateral side (red and blue arrows).

**Fig. 3:**
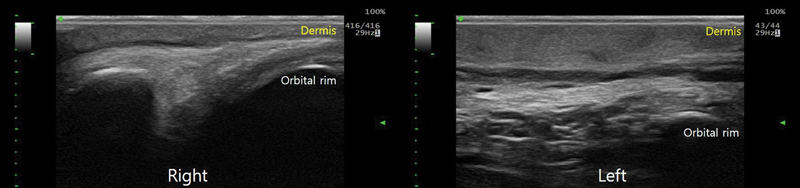
Ultrasonographic findings of a patient with left upper eyelid lymphedema showing thickened dermis and a coarse subcutaneous layer above the orbital rim.

The computed tomography scan showed diffuse vague swelling compared to the contralateral side without any mass-like lesions such as lymphatic malformations ( Fig. 2 ). In addition, ultrasonography revealed severe soft tissue thickening between the dermis and orbital rim compared with the unaffected right side ( Fig. 3 ). Preoperative indocyanine green (ICG) lymphography was performed by injecting 0.1 mL of ICG at medial, central, and lateral side above the left eyebrow which resulted in focal severe dermal backflow in the affected area and normal lymphatic drainage from the malar area.

**Fig. 4:**
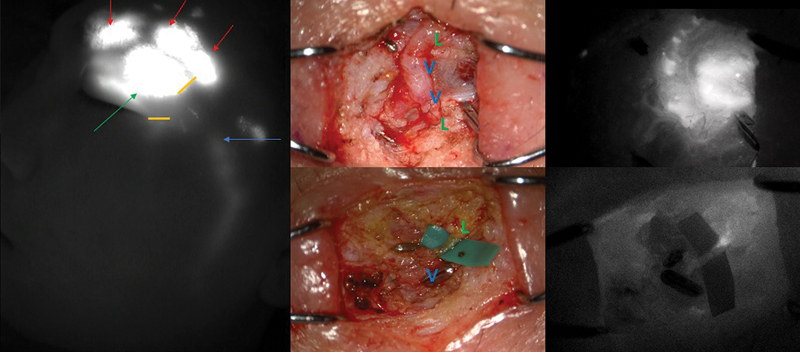
(Left) Preoperative indocyanine green (ICG) lymphography after injection of ICG above left eyebrow (red arrow) showing severe dermal backflow on the affected area (green arrow) and normal lymphatic drainage (blue arrow) from the malar area. (Center) Lymphovenous anastomoses were performed at the lateral eyebrow and lateral canthal area by separate incisions (yellow line) and (right) the patency was confirmed by intraoperative ICG lymphography. (L, lymphatic vessel; V, vein).

**Fig. 5:**
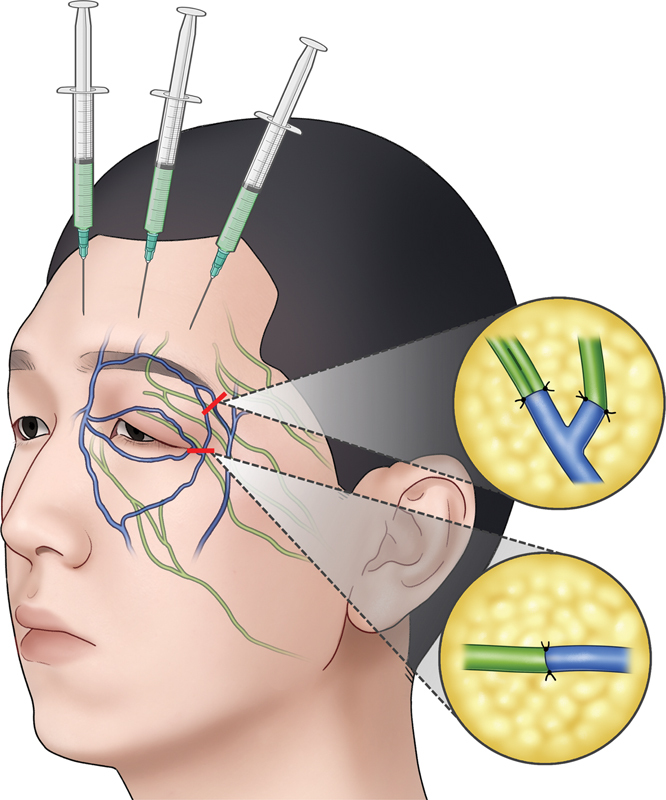
Schematic illustration demonstrating the anatomical basis of periorbital lymphatics and veins for lymphovenous anastomosis.

**Fig. 6:**
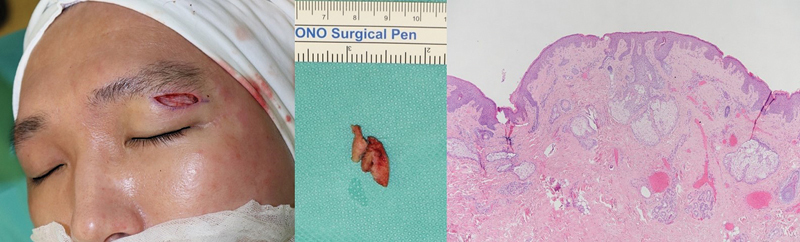
Intraoperative findings of subbrow fibrous tissue excision (left, center) and its histopathologic findings demonstrated a thickened epidermis and dermis with lymphocyte dominancy and scanty adipose tissue (right).

**Fig. 7:**
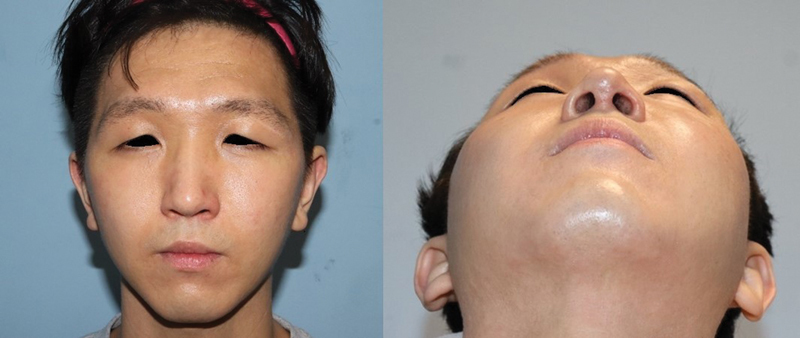
Postoperative clinical photograph at 2 years postoperatively showing improvement of left upper eyelid lymphedema.

Under general anesthesia, LVA was performed on the lateral canthal area and lateral eyebrow in the area of dermal backflow through two separate 1-cm-sized transverse incisions targeted for eyelid and frontal branches of facial lymphatic vessels ( Fig. 4 ). After careful dissection in the subcutaneous layer where very little fat was left and mostly substituted by fibrous tissue, dilated ectatic lymphatic vessels were found. A total of four LVAs were performed between 0.6-, 0.5-, 1.0-, and 0.4-mm-sized lymphatics and 1.6-, 1.5-, and 0.5-mm-sized veins in an end-to-end fashion ( Figs. 4 and 5 ). In addition, a 1.5 × 0.3-cm-sized thick fibrous tissue along the subbrow area was excised according to patient requirements ( Fig. 6 ). These histopathological findings demonstrated a thickened epidermis and dermis with scanty adipose tissue, where retro-orbicularis oculi fat was replaced by fibrous and lymphocyte dominant inflammatory tissue. The symptoms improved significantly 2 years postoperatively, and the patient was satisfied with the results without recurrence ( Fig. 7 ).

## Discussion

Traditionally, eyelid lymphedema has been treated by surgical debulking and excision, followed by skin grafting in cases of large-area invasion. However, the results were aesthetically poor, and more importantly, recurrence was reported in most cases. 5 11 12 To prevent recurrence and achieve complete resolution, pharmacological options such as minocycline, dexamethasone, and prednisolone have been attempted but failed. 3 9

Recently, Koshima et al introduced the concept of supermicrosurgery, which utilizes lymphatics and venules smaller than 0.8 mm and has been applied in lymphedema treatment. 13 Since then, many reports have demonstrated the effect of supermicrosurgical treatment, mainly in secondary lymphedema of the upper and lower extremities. Although rare, there are a few reports on the application of LVAs in facial lymphedema. Most cases involved secondary lymphedema related to head and neck cancer, for which neck dissection and radiotherapy were performed previously. 14 15 16

To understand facial lymphedema, the anatomy of facial lymphatic vessels should be comprehended. Generally, frontal, eyelid, nasal, oral, and mental branches from cranial to caudal are responsible for facial lymphatic drainage. 17 Currently, investigations for diagnostic tools to indicate impaired facial lymphatic vessels are rarely reported. Only a few reports regarding lymphoscintigraphy and ICG lymphography for facial lymphedema have been published but different contrast injection sites were selected in each report. For lymphoscintigraphy, injection of radiocolloids at the level of the upper lip and glabella was suggested. 18 19 Injection sites for ICG were reported as median lines including upper and lower lip, glabella, and frontal region. 20 In our case, since lymphedema manifested focally on the left lateral upper eyelid and eyebrow, we injected ICG on the frontal area just above the eyebrow to efficiently visualize frontal and eyelid branches around the impeded area. We could also visualize normal lymphatic drainage on the medial half of the upper eyelid by this method and therefore, the frontal area above the eyebrow seems to be an appropriate injection site for the diagnosis of upper eyelid lymphedema.

Interestingly, all the cases reported that LVA was performed in the preauricular area. The preauricular region is the area where lymphatic vessels from the upper and midface merge and drain into the lymph nodes (LNs). 21 In particular, the frontal branches pass lateral to the lateral eyebrow and drain into the preauricular and/or deep parotid LNs, and the outer eyelid branches drain into the mandibular LNs and/or infra-auricular LNs. Furthermore, the superficial temporal vein runs parallel to these lymphatics consistently in the preauricular area; therefore, it is one of the most suitable regions for surgeons to perform LVA without anatomical concerns.

However, in the case of eyelid lymphedema, the preauricular area is not the exact area that is affected by the disease. In other words, performing LVA in this region may pose a risk of disturbing normal healthy lymphatics because we need to cut or create a hole to connect a vein. Although previous reports have demonstrated improvement in symptoms postoperatively, the authors thought it would be more logical to perform LVA in areas where lymphedema manifested. Therefore, we attempted to identify the distal lymphatics in the area where the dermal backflow was visualized. In this case, dermal backflow extended from the lateral half of the upper eyelid to the lateral canthal and lateral eyebrow areas, and LVAs were performed according to the anatomical pathway in which the outer eyelid and frontal branches of the lymphatic vessels pass through these areas. These lymphatics were targeted, and the superior and lateral palpebral veins were used as recipients. Since the lymphatics were ectasis type and dilated from 0.4 to 0.6 mm in diameter, it was feasible to perform LVA to as large as 1.6-mm-sized veins.

Treatment options are very limited for eyelid lymphedema compared to typical lymphedema in the extremities. Vascularized LN transfer may result in a very large scar, and liposuction is difficult to perform in shallow and small areas. Conservative treatment, including constant compression therapy, is impossible before or after any type of debulking surgery, and there is a high chance of recurrence. Therefore, LVA is the most suitable treatment option for eyelid lymphedema with a short scar and satisfactory outcomes. By understanding the anatomy of the lymphatics and veins in the periorbital area, LVA can be performed readily and constantly using supermicrosurgery.
